# The Role of Complement Anaphylatoxins (C3a and C5a) in Non-small Cell Lung Cancer: A Systematic Review

**DOI:** 10.7759/cureus.105423

**Published:** 2026-03-18

**Authors:** Konstantinos Skarentzos, Despoina Kyriaki Karagialani, Vangelis Mitros, Panagiota Economopoulou, Periklis Foukas

**Affiliations:** 1 Second Department of Pathology, National and Kapodistrian University of Athens, Medical School, "Attikon" University Hospital, Athens, GRC; 2 Medical School, National and Kapodistrian University of Athens, Athens, GRC; 3 Medical Oncology Unit, Second Department of Internal Medicine - Propaedeutic, National and Kapodistrian University of Athens, Medical School, “Attikon” University Hospital, Athens, GRC

**Keywords:** anaphylatoxins, c3a, c5a, nsclc, systematic review

## Abstract

The complement anaphylatoxins C3a and C5a are critical effectors of innate immunity. Beyond this role, emerging evidence has implicated them in cancer progression. However, their specific functions in non-small cell lung cancer (NSCLC) have not been systematically reviewed. This review aimed to synthesize the evidence on the contribution of C3a and C5a to NSCLC progression and their potential as therapeutic targets. This systematic review followed PRISMA (Preferred Reporting Items for Systematic Reviews and Meta-Analyses) guidelines. A comprehensive search of PubMed, Scopus, and Cochrane Library was performed up to May 2025. The PICO (Population, Intervention, Comparison, Outcome) framework guided the inclusion of studies involving NSCLC patients or human cell lines, reporting on C3a/C5a levels and their association with clinical outcomes or pro-tumoral effects. The risk of bias was assessed using the OHAT tool. From 11,838 initially identified records, six studies met the inclusion criteria based on PICO criteria requiring direct investigation of C3a/C5a in human NSCLC contexts. The evidence strongly and consistently points to a critical pro-tumoral role for the C5a/C5aR1 axis. Key findings include: C5a promotes NSCLC cell proliferation, migration, invasion, and epithelial-to-mesenchymal transition; high C5aR1 expression is an independent prognostic factor for worse recurrence-free survival; and tumor-derived C5a enhances angiogenesis and metastatic potential, particularly to bone. NSCLC cells evade complement attack by producing regulators like Factor H. In contrast, the role of C3a was less defined. While C5a was consistently elevated and active in the tumor microenvironment, one study noted a local decrease of C3a in tumor tissues, suggesting a potentially different or context-dependent function. Risk of bias assessment using the OHAT tool indicated low to moderate risk across the included studies. This systematic review shows that complement anaphylatoxins, especially C5a, drive NSCLC progression by promoting a pro-tumoral microenvironment and metastasis. Targeting the C5a/C5aR1 axis is a promising therapeutic strategy, though more research is needed.

## Introduction and background

The introduction of immunotherapy, specifically drugs that block the PD-1/PD-L1 pathway, has revolutionized the first-line treatment landscape for patients with advanced non-small cell lung cancer (NSCLC) without targetable driver mutations. Therapeutic approaches with immune checkpoint inhibitors, either alone or in combination with chemotherapy, represent the standard of care now, especially for tumors with high PD-L1 expression on tumor cells. While this strategy has improved survival outcomes, its efficacy is limited by the fact that many patients eventually develop resistance to the treatment. Therefore, a critical focus of ongoing research is to decipher the mechanisms behind this resistance and to create new therapeutic approaches, including combination therapies and predictive biomarkers, to help more patients achieve long-term remission [1]. A deeper understanding of the immune status within the tumor microenvironment may lead to higher response rates and fewer adverse events.

The complement system, a key component of the innate arm of the immune system, plays an important role in shaping adaptive immune responses. This is originally considered as an immune mechanism exerting anti-tumor activity by promoting the formation of the membrane attack complex (MAC) to kill tumor cells. However, studies that integrate its role in the tumor microenvironment have shown that it actually promotes tumor growth via certain complement proteins, such as the C3a and C5a anaphylatoxins, that may help tumor cells to escape the body’s immunosurveillance mechanisms. Although under normal circumstances the liver is the main source of complement protein production, recent data suggest that cancer cells, and especially those of NSCLC, can produce those proteins locally [2]. NSCLC exploits complement-derived proteins in many ways in order to evade immunity and grow. The complement system is activated through three main pathways (classical, alternative, and lectin), all converging on the cleavage of C3 and C5 to generate the anaphylatoxins C3a and C5a. These fragments bind to their respective receptors (C3aR and C5aR1) on immune and tumor cells, triggering pro-inflammatory and immunomodulatory effects that can be co-opted by tumors to promote progression. Specifically, tumor cells express factor H on their cell membranes, which helps them avoid the activation of complement and their subsequent lysis [3]. Factor H functions as a crucial complement regulator by suppressing the alternative pathway, which ultimately limits the generation of the pro-inflammatory anaphylatoxins C3a and C5a and their subsequent signaling through receptors C3aR and C5aR [4]. Furthermore, it was shown that NSCLC tumor cells promote metastasis not only by producing the complement component C5a, which activates the C5aR1 receptor but also by expressing this receptor. This signaling enhances tumor invasiveness and migration, contributing to skeletal metastasis in lung cancer [5].

However, while the pro-tumoral role of C5a in NSCLC has been increasingly recognized, the specific contribution of C3a remains poorly understood. It is unclear whether C3a functions similarly to C5a, plays a distinct or even opposing role, or is simply a bystander in the tumor microenvironment. This uncertainty represents a significant gap in the literature.

Despite these emerging findings, the specific roles of C3a and C5a in NSCLC have not been systematically synthesized, and no existing review has comprehensively evaluated the clinical and experimental evidence linking these anaphylatoxins to tumor progression. Notably, several complement inhibitors, including those targeting C5a or its receptor, are already in clinical use for other diseases, highlighting their potential as new therapeutic options for NSCLC.

The aim of this systematic review is to synthesize the evidence on the contribution of the anaphylatoxins C3a and C5a to NSCLC progression and their potential as therapeutic targets. To our knowledge, this is the first systematic review to address this specific subject.

## Review

Methods

Eligibility Criteria

This systematic review of the literature was performed according to the PRISMA guidelines [6]. The review protocol was registered with the Open Science Framework [7].

The PICO (Population, Intervention, Comparison, Outcome) framework was used to define the scope. The Population consisted of NSCLC patients and human NSCLC cell lines. The Intervention/Exposure was the presence or level of C3a and/or C5a. Comparisons included: (i) NSCLC patients versus healthy subjects, and (ii) human NSCLC cell lines versus normal human bronchial epithelial cell lines. To minimize therapy-related bias, only studies involving treatment-naïve patients (those who had not received any clinical treatment prior to sample collection) were included. The primary Outcomes for clinical studies were overall survival, progression-free survival, and other clinical outcomes related to C3a/C5a. For studies using cell lines, outcomes of interest included tumor cell growth, invasiveness, and other phenotypic features in relation to C3a/C5a levels in the tumor supernatant microenvironment.

Exclusion criteria were as follows: studies not published in English; animal studies; studies using non-human or non-NSCLC cell lines; systematic reviews, literature reviews, case reports, and case series; and non-peer-reviewed publications (e.g., conference proceedings, preprints).

Information Sources and Search Strategy

A comprehensive search of the PubMed, Scopus, and Cochrane Library databases was performed using the following query: “((C3a OR C5a) OR Complement) AND (NSCLC OR (Non-small AND cell AND lung AND cancer) OR adenocarcinoma OR (squamous AND cell AND carcinoma) OR SCC OR (lung AND cancer))”. The use of three bibliographic databases and a broad search strategy was intended to maximize the retrieval of relevant articles.

Selection Process

Two reviewers (D.K.K. and V.M.) independently screened titles and abstracts against the eligibility criteria, blinded to each other's decisions. Conflicts were resolved through discussion. The same two reviewers then independently conducted a full-text assessment of the remaining articles. A consensus was reached for all included studies, and reasons for exclusion at the full-text stage were documented.

Data Collection Process and Data Items

Data were extracted independently by two reviewers (K.S. and D.K.K.) using a standardized form. Consensus was reached via discussion. Extracted data items included: first author, year of publication, country, patient and control group demographics, lung cancer type and stage, overall survival, disease-free survival, progression-free survival, cell line details (name, origin, type), C3a and C5a levels (in plasma, tissue, or cell culture), and experimental methodologies.

Data Synthesis

Due to substantial differences in study design, outcome measures, and patient populations across the included studies, a narrative synthesis approach was employed. Clinical and experimental evidence were synthesized separately: clinical findings were summarized to assess associations between C3a/C5a levels and patient outcomes, while in vitro findings were synthesized to elucidate underlying mechanisms of C3a/C5a function in NSCLC cell lines. Within each category, results were organized thematically based on the specific role of C3a or C5a investigated. Quantitative meta-analysis was not feasible due to methodological heterogeneity and the limited number of studies.

Risk of Bias Assessment

The quality of the included studies was assessed independently by two authors (K.S. and V.M.), who were blinded to each other's assessments. (Office of Health Assessment and Translation) Risk of Bias Tool was used for human studies [8], and a modified version of the OHAT tool was applied to in vitro studies [9]. This tool comprises 9-11 questions, each rated on a scale from "definitely low" to "definitely high" risk of bias. Any discrepancies in ratings were resolved through discussion.

Software

EndNote™ 20 was used as citation manager [10].

Results

Study Selection

**Figure 1:**
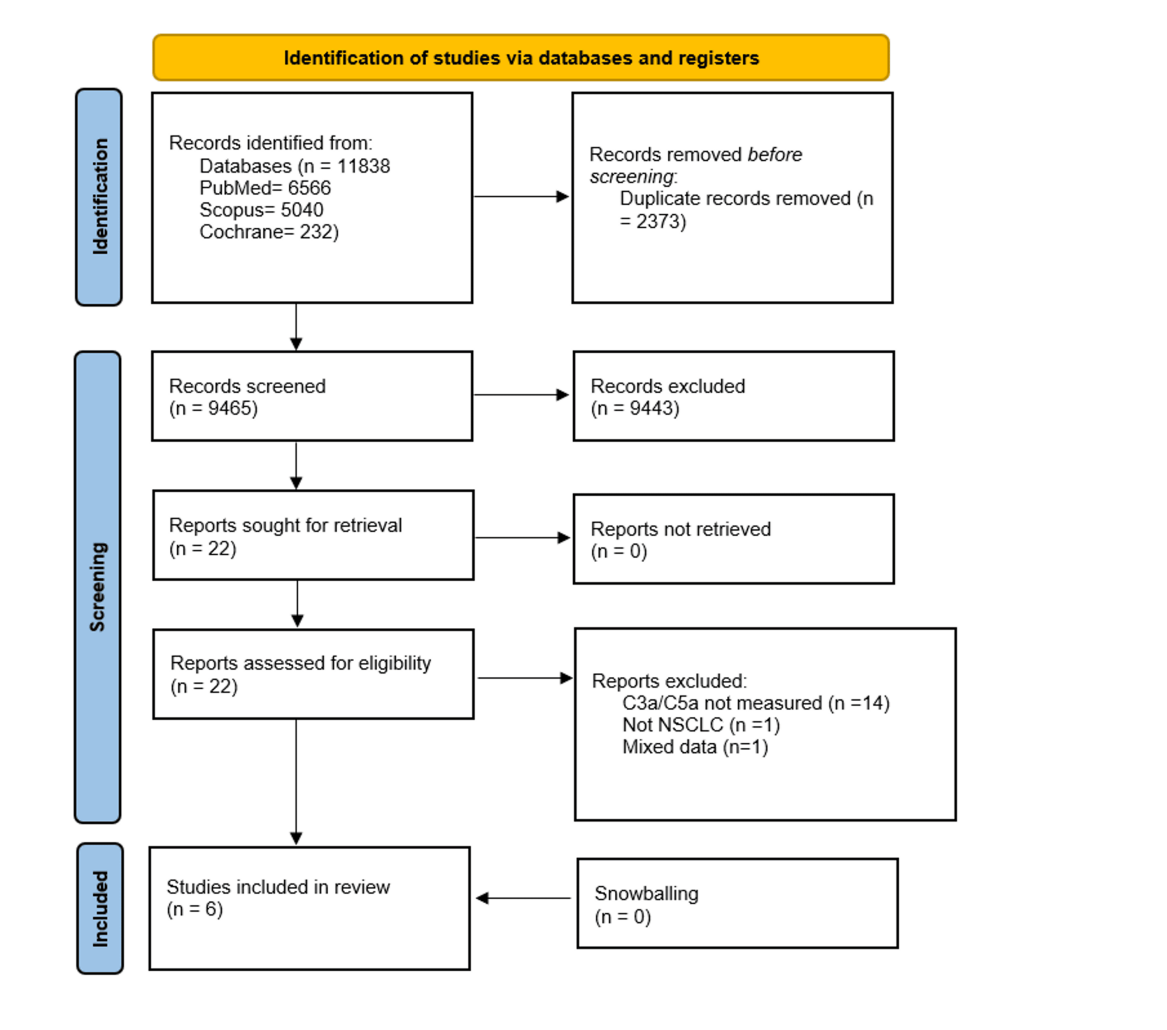
PRISMA Flow Diagram PRISMA: Preferred Reporting Items for Systematic Reviews and Meta-Analyses

A total of 11,838 articles were identified through database searching (PubMed: n=6,566; Scopus: n=5,040; Cochrane: n=232) (Figure 1). After removing 2,373 duplicate records, 9,465 records underwent title and abstract screening. Twenty-two articles were selected for full-text retrieval. Of these, 14 were excluded because C3a/C5a was not measured, one was ineligible as it was not related to NSCLC, and another was excluded for reporting mixed data on both NSCLC and small cell lung cancer. A manual search of reference lists (snowballing) yielded no additional results. Consequently, six articles were included in this systematic review [3,5,11-14].

Study Characteristics and Results of Individual Studies

The characteristics and key findings of the included clinical studies are summarized in Table 1, while Table 2 provides an overview of the experimental studies, detailing the cell lines used and the main outcomes related to C3a and C5a function.

**Table 1:** Clinical Studies NSCLC: Non-small cell lung cancer; —: not applicable.

Authors	C3a	C5a	Patients (n)	Control (n)	Important outcomes
Ajona et al. [5]	No	Yes	95	—	Patients with high C5aR1 expression had statistically significant decreased recurrence-free survival
Corrales et al. [11]	No	Yes	50	50	C5a plasma levels were significantly increased in patients versus healthy individuals
Luo et al. [12]	Yes	Yes	94	102	High C5a expression, but no C3a expression
Zhao et al. [13]	No	Yes	52	—	Increased plasma level of C5a and increased expression of proliferation-related genes
Zhao et al. [14]	Yes	No	20	—	Decreased C3a levels in NSCLC tissues

**Table 2:** Experimental Studies NSCLC: Non-small cell lung cancer; MMP: metalloproteinase

Authors	C3a	C5a	Malignant cell lines	Important outcomes
Ajona et al. [3]	Yes	Yes	H1264 and A549	Inhibition of factor H increased significantly the release of C5a in cancer cells
Ajona et al. [5]	No	Yes	A549M1 and H460M5	C5a led to increased levels of IL-8, monocyte chemotactic protein 1, and vascular endothelial growth factor; C5a enhanced cancer cell motility, invasiveness, and MMP activity, and reduced cell-matrix adhesion
Corrales et al. [11]	No	Yes	A549, H23, H157, H460, H1299, HTB58 and H727	C5a was produced in all cell lines except H727, whereas no production of C5a was found in normal bronchial epithelial cell lines
Luo et al. [12]	Yes	Yes	A549	C5a induced cancer cell proliferation, invasion, adhesion and epithelial-to-mesenchymal transition of tumor cells
Zhao et al. [13]	No	Yes	A549, H1299, H1975, and H1703	C5a induced cancer cell proliferation; C5a binding to C5aR was responsible for upregulation of proliferation-related gene expression
Zhao et al. [14]	Yes	No	A549	Co-culture of A549 and hepatocytes (QSG-7701) produced high level of complement proteins

In the experiment by Ajona et al., the H1264 (lung adenocarcinoma) and A549 (bronchoalveolar lung carcinoma) cell lines were used. This study focused on the role of the complement regulators factor H, CD46, and CD55 in blocking complement activation. The results demonstrated that inhibition of factor H significantly increased C5a release in both H1264 and A549 cells, whereas blockade of CD46 and CD55 had no effect in C5a levels. These findings suggest that the expression of the complement inhibitor factor H by lung cancer cells can prevent complement activation and complement mediated tumor-cell lysis [3]. Consequently, decreased C5a levels could serve as a potential marker of factor H activity, which may be associated with increased tumor growth.

In another study [5], the role of the C5a/C5aR1 axis (where C5aR1 is the receptor for the cleaved complement component C5a) was investigated in 95 NSCLC patients from two cohorts and in cell lines (A549M1 and H460M5). Patients were stratified based on C5aR1 expression levels. Those with high C5aR1 expression had a statistically significant decrease in recurrence-free survival. Overall survival was also reduced in the high C5aR1 group, although this difference was not statistically significant. A multivariable analysis confirmed that high C5aR1 expression was an independent prognostic factor for shorter recurrence-free survival. In the in vitro experiments, incubation of A549 cells with C5a led to increased levels of IL-8, monocyte chemotactic protein 1, matrix metalloproteinase activity, and vascular endothelial growth factor. Furthermore, C5a was shown to induce lung cancer cell motility and invasiveness while reducing cell-matrix adhesion, without affecting cell growth kinetics [5]. These findings further demonstrate that C5a production may enhance the invasiveness and metastatic potential of tumor cells.

In the study by Corrales et al., the researchers utilized lung cancer cell lines from the American Type Culture Collection and plasma samples from 50 NSCLC patients and 50 healthy individuals matched by age, sex, and smoking history. C5a plasma levels were significantly higher in NSCLC patients compared to healthy controls (p<0.001), whereas no statistically significant differences in C5a levels were observed between adenocarcinomas and squamous cell carcinomas or across different cancer stages. All evaluated lung cancer cell lines (A549, H23, H157, H460, H1299, HTB58), with the exception of H727, produced C5a after 24 hours of culture in serum-free medium [11]. These results indicate that tumor cells themselves are a source of detectable C5a in the plasma of NSCLC patients.

Luo et al. collected malignant pleural effusions (MPE) from 76 patients with lung adenocarcinoma and 18 with squamous cell carcinoma. They measured C3a and C5a levels in the malignant pleural exudates, detecting high C5a expression which was attributed to activation of the classical and alternative complement pathways. In contrast, C3a was not detected. The study also demonstrated that C5a correlated with the recruitment of CD14+ and CD16+ monocytes and increased IL-1β release from these cells within the MPE. In their experimental setting, by using the A549 cell line, C5a was found to induce cancer cell proliferation, promote invasion, enhance adhesion, and stimulate epithelial-to-mesenchymal transition (EMT). More specifically, the Ki-67 proliferation index was elevated following C5a stimulation. In the same line, C5a-treated cells also showed increased expression of intercellular adhesion molecule-1 (ICAM-1), leading to enhanced cellular adhesion. Furthermore, treatment with C5a significantly increased the expression of mesenchymal markers (N-cadherin and vimentin) while the epithelial marker E-cadherin was decreased, confirming the promotion of EMT [12].

In another study, 52 fresh NSCLC tissue samples and paired adjacent normal tissues were acquired from 52 patients who had not received any preoperative chemotherapy or radiotherapy. Human NSCLC cell lines (A549, H1299, H1975, and H1703) were also utilized. The researchers measured mRNA levels of proliferation-related genes in the fresh NSCLC tissues and found them to be significantly elevated compared to normal tissues. Correspondingly, plasma C5a levels were markedly increased in the same patients. Furthermore, an experiment using human cancer cell lines (A549 and PC9) was conducted to investigate the association between C5a concentration and the expression of the proliferation-related genes KLF5, GCN5, and GDF15. The results confirmed that higher C5a levels were correlated with increased expression of these genes. It was also demonstrated that this upregulation is dependent on C5a binding to its receptor, C5aR [13].

Zhao et al. obtained 20 cancer tissues and 20 paired adjacent normal lung tissues from treatment-naïve adenocarcinoma patients. Using an iTRAQ proteomic approach, they identified a decrease in the expression of 31 complement and complement-related proteins, including C3a, in the tumor tissues compared to the normal tissues (C5a was not investigated). Given that the liver is the primary site of complement protein synthesis, the researchers conducted a co-culture experiment using NSCLC cells (A549), human hepatocytes (QSG-7701), and normal human bronchial epithelial cells. They reported that the co-culture of A549 cells with QSG-7701 hepatocytes resulted in higher complement protein production compared to A549 monoculture, QSG-7701 monoculture, or co-culture of hepatocytes with normal bronchial epithelial cells [14].

Integrated Summary of Findings

Across the six included studies, a consistent biological signal emerges regarding the pro-tumoral role of the C5a/C5aR1 axis in NSCLC. Clinical evidence demonstrates that elevated C5a plasma levels [11,13] and high C5aR1 expression [5] are associated with worse patient outcomes, including decreased recurrence-free survival. This is supported by experimental findings showing that C5a promotes key oncogenic processes across multiple NSCLC cell lines, including proliferation [12,13], migration and invasion [5,12], epithelial-to-mesenchymal transition [12], and angiogenesis [11]. Mechanistically, C5a signaling appears to be mediated through upregulation of proliferation-related genes (KLF5, GCN5, GDF15) [13], increased expression of adhesion molecules (ICAM-1) [12], and enhanced metalloproteinase activity [5]. In contrast, evidence for C3a is limited and inconsistent: one study detected no C3a in malignant pleural effusions [12], while another study found decreased C3a levels in tumor tissues [14], suggesting a potentially distinct or context-dependent role that requires further investigation.

Risk of Bias

Overall, all articles were characterized by low risk of bias. For studies involving both patient cohorts and in vitro experiments, the risk of bias was assessed separately for each component, as certain OHAT tool questions are specific to clinical or experimental designs. Consequently, Table 3 presents two distinct risk-of-bias assessments for these studies.

**Table 3:** Risk of Bias Q1: Was administered dose or exposure level adequately randomized? Q2: Was allocation to study groups adequately concealed? Q3: Did selection of study participants result in the appropriate comparison groups? Q4: Did study design or analysis account for important confounding and modifying variables? Q5: Were experimental conditions identical across study groups? Q6: Were research personnel blinded to the study group during the study? Q7: Were outcome data complete without attrition or exclusion from analysis? Q8: Can we be confident in the exposure characterization? Q9: Can we be confident in the outcome assessment (including blinding of assessors)? Q10: Were all measured outcomes reported? Q11: Were there no other potential threats to internal validity? "++" = definitely low risk of bias; "+" = probably low risk of bias; "-" = probably high risk of bias/not reported; NA = not applicable.

Authors	Type of study	Q1	Q2	Q3	Q4	Q5	Q6	Q7	Q8	Q9	Q10	Q11
Ajona et al. [3]	Experimental	++	++	NA	NA	++	-	++	++	-	++	+
Ajona et al. [5]	Human	NA	NA	++	++	NA	NA	++	++	++	++	+
Ajona et al. [5]	Experimental	++	++	NA	NA	++	-	++	++	-	++	+
Corrales et al. [11]	Human	NA	NA	++	++	NA	NA	++	++	-	++	+
Corrales et al. [11]	Experimental	++	++	NA	NA	++	-	++	++	+	++	+
Luo et al. [12]	Human	NA	NA	++	++	NA	NA	++	++	+	++	+
Luo et al. [12]	Experimental	++	++	NA	NA	++	-	++	++	+	++	+
Zhao et al. [13]	Human	NA	NA	++	++	NA	NA	++	++	+	++	+
Zhao et al. [13]	Experimental	++	++	NA	NA	++	-	++	++	+	++	+
Zhao et al. [14]	Human	NA	NA	++	++	NA	NA	++	++	+	++	+
Zhao et al. [14]	Experimental	++	++	NA	NA	++	-	++	++	+	++	+

For the in vitro components, questions 3 and 4 were marked as not applicable, as they pertain to participant selection and confounding variables not relevant to experimental designs. Experimental studies received "definitely low" risk ratings for questions 1, 2, and 5, reflecting the use of homogenous cell lines and standardized conditions. However, all experimental studies received "probably high" risk for question 6 due to lack of reported blinding techniques or use of automated systems [9].

For human study components, questions 1, 2, 5, and 6 were deemed not applicable, as these relate to experimental conditions not relevant to cohort or case-control designs [8].

Discussion

NSLC cells utilize several mechanisms to evade complement activation. These include expressing complement regulators such as Factor H, CD55 (decay-accelerating factor), and CD46 (membrane cofactor protein), which protect tumor cells from complement-dependent cytotoxicity. In vitro studies have demonstrated that NSCLC cells produce Factor H, which effectively inhibits complement activation. Further investigation into the role of these regulators in the classical pathway revealed that inhibiting Factor H with a specific antibody (mAb OX-24) significantly increased C5a release in tumor cell lines. In contrast, the blockade of CD46 and CD55 did not affect C5a release [3]. Since C5a serves as a marker for C5 convertase activity, this suggests that evasion mechanisms which block the C5 convertase, such as Factor H secretion by tumor cells, could be detected by monitoring C5a levels. Therefore, measuring C5a in patient serum or the tumor microenvironment could be a useful indicator of C5 convertase activity and potential complement inhibition.

The role of the C5a/C5aR1 axis was investigated in a cohort of 95 NSCLC patients, stratified based on C5aR1 expression, and in NSCLC cell lines [5]. Given that bones are the most frequent site of metastasis in NSCLC, the complement system was hypothesized to play a significant role in this process. The study identified C5aR1 as a key mediator, as significantly higher expression was found in patients with osseous metastases compared to those with metastases in other organs. This finding was supported by experiments involving the activation of C5a, which upon binding to the C5aR1 receptor, increased the activity of metalloproteinases (MMPs). However, as these experiments were conducted in cell lines, they cannot fully represent the complexity of tumor biology in human patients, including interactions with immune cells, stromal components, and systemic factors. This enhancement led to increased cell migration, invasion, and osteolysis. Furthermore, receptor activation was shown to facilitate tumor progression by inhibiting T-cell function, subsequently leading to the secretion of immunoregulatory mediators such as TGF-β and IL-1β. Conversely, inhibition of C5aR1 reduced osteoclast activity and minimized cell migration, invasion, and MMP activity in A549M1 cells. Collectively, these findings suggest that blocking the C5a/C5aR1 axis negatively affects bone metastasis in NSCLC and could potentially serve as a novel therapeutic target to reduce the metastatic potential of the disease [5].

Corrales et al. investigated the role of the C5a anaphylatoxin in lung cancer patients [11]. Measurements of C5a levels in serum revealed significantly higher concentrations in patients versus healthy participants. Notably, there was no variation in C5a levels among different cancer types or stages. Furthermore, C5a was found to contribute to tumor progression by promoting angiogenesis, as evidenced by enhanced cell migration and tube-like structure formation [11]. These findings point to promising new therapeutic strategies, either by targeting C5a signaling or tumor-derived proteases individually, or by combining both approaches to more effectively inhibit C5a-driven angiogenesis and tumor growth. In contrast, the role of C3a in NSCLC remains poorly defined, with limited and inconsistent data across studies, underscoring the need for further investigation.

Luo et al. detected high C5a levels in malignant pleural effusions, suggesting complement activation contributes to this poor prognostic feature [12]. The study detected high levels of the C5a complement protein in MPE, suggesting activation of the complement pathway. C5a acts as a potent chemoattractant for CD14+ and CD16+ monocytes, recruiting them to inflamed tissues. Furthermore, C5a triggers IL-1β release, which promotes the formation of malignant pleural effusion. Using A549 cancer cell lines, the researchers observed that C5a promoted cell proliferation (as indicated by elevated Ki-67 levels) and enhanced tumor adhesion via increased ICAM-1 expression. The study also demonstrates that the C5a/C5aR axis plays a protective role for cancer cells, helping them resist DDP (cisplatin)-induced apoptosis. Interestingly, cancer cells not only evade this immunosurveillance mechanism but also exploit it to enhance migration, invasion, intercellular adhesion, and epithelial-mesenchymal transition. Pharmacological blockade of C5aR or inhibition of IL-1β signaling was shown to reduce monocyte activity and limit tumor progression in the pleural cavity. This study suggests that targeting the C5a and IL-1β pathways may offer new therapeutic strategies to restrict tumor growth [12].

Zhao et al. identified a C5a-KLF5-GCN5-GDF15 signaling axis driving proliferation, revealing a novel downstream pathway in NSCLC [13]. All patients included in the study had not undergone any preoperative chemotherapy or radiotherapy. Additionally, human NSCLC cell lines (A549, H1299, H1975, and H1703) were utilized for in vitro analyses. The researchers investigated the role of C5a in promoting proliferation by examining the KLF5, GCN5, and GDF15 genes, which are associated with the C5a signaling mechanism. These genes were analysed following the induction of NSCLC cell proliferation by C5a stimulation both in vivo and in vitro. Experiments in NSCLC cell lines revealed elevated expression of KLF5, GCN5, and GDF15 after stimulation with the C5a protein. Overall, activation of the C5a-KLF5-GCN5-GDF15 pathway enhanced the proliferation of A549 cells. The results indicate that the C5a-induced activation of this pathway is essential for the growth of NSCLC cells and could represent a potential target for new treatments [13].

Another study investigated complement-related proteins by analyzing 20 cancer tissues and 20 adjacent normal lung tissues from 20 treatment-naïve lung adenocarcinoma patients [14]. Using an iTRAQ proteomic approach, the study focused on the correlation of these proteins, specifically emphasizing C3a over C5a. The findings revealed a significant imbalance in the lung cancer microenvironment regarding complement system function. Complement proteins, including C3, were decreased locally within the tumor tissues, potentially impairing the immune attack against malignant cells. Simultaneously, the liver, as the primary source of C3 production, exhibited a compensatory mechanism that increased C3 plasma levels in response to tumor-derived signals. This systemic-plasma imbalance demonstrates how lung cancer may simultaneously weaken local immune responses while promoting systemic inflammation [14]. A deeper understanding of this process could offer new opportunities for novel therapies and biomarker development in lung cancer.

Following an extensive systematic review of the existing literature on the role of the complement system in NSCLC, it is concluded that this area requires further research. Although the complement system plays an important role in lung cancer progression, the exact mechanisms involved have not been thoroughly elucidated. A precise understanding of these mechanisms is essential for the development of new treatments. Promisingly, several complement-targeted therapies, especially those aimed at C5a or its receptor, are currently under investigation. A foundational drug in this class is eculizumab, an anti-C5 monoclonal antibody that inhibits the cleavage of C5 into C5a and C5b. By preventing C5b formation, eculizumab inhibits the formation of the MAC, which is responsible for cell lysis and contributes to systemic inflammation. However, these agents are currently approved only for non-oncological diseases, and their therapeutic relevance in NSCLC remains uncertain pending dedicated clinical trials.

After a thorough review, the studies included in this systematic review each have certain limitations, which will be addressed in the following section. For instance, the findings of one study were based on experiments using only two cell lines (H1264 and A549) and animal models, as patient-derived samples were not included [3]. Zhao et al. collected NSCLC tumor tissues and paired adjacent healthy tissues from 52 patients; however, the study lacked a control group of healthy individuals without NSCLC [11]. Another study used 20 paired samples of NSCLC tissues and their matched adjacent normal tissues. The relatively small sample size and the lack of a healthy control group may impact the significance of the findings [14].

Some limitations of our study must be acknowledged. Although a thorough screening process and a broad search algorithm were used in this systematic review (11,838 articles screened), only a few studies met our inclusion criteria. The small number of eligible studies limits the strength of our conclusions, and the limited sample sizes within the included studies further reduce statistical power and generalizability, highlighting the need for further research in this area. Given this small and heterogeneous evidence base, mechanistic conclusions should be considered preliminary and interpreted with caution. Our search strategy focused on three major biomedical databases (PubMed, Scopus, and the Cochrane Library), which collectively provide extensive coverage of the literature in this field; however, we cannot exclude the possibility that relevant studies indexed in other databases or sources may not have been identified. In addition, grey literature (e.g., conference proceedings, preprints, unpublished data) was not searched, and publication bias cannot be ruled out, as studies with positive or significant findings may be more likely to be published than those with null or negative results. Additionally, the evidence regarding the role of C3a in NSCLC was limited and inconsistent across the included studies, precluding definitive conclusions about its contribution to tumor progression. Furthermore, significant methodological heterogeneity was observed across studies, including differences in experimental models, measurement techniques, outcome variables, and patient populations. This heterogeneity was particularly pronounced between clinical and experimental studies, limiting our ability to integrate findings across these different study types. We also acknowledge that animal studies were excluded from this review based on our eligibility criteria. While this decision was made to maintain focus on human-relevant evidence and avoid interspecies variability in complement biology, we recognize that including animal models could have provided additional insights into tumor-immune interactions and metastatic mechanisms. Importantly, the included studies have not consistently accounted for potential confounding variables such as tumor stage, histological subtype, treatment history, PD-L1 expression status, and patient comorbidities-factors that could influence complement activation and independently affect patient outcomes. Consequently, a quantitative meta-analysis was not feasible, and this review provides only a qualitative synthesis of the findings. Without pooled statistical analysis, we cannot estimate the magnitude of the effect of C3a/C5a signaling on NSCLC outcomes or formally assess statistical heterogeneity across studies. Finally, the restriction to studies published in English may have excluded potentially relevant research published in other languages.

Regarding the future implications of this research, several complement inhibitors are already approved for diseases such as myasthenia gravis [15] and glomerular diseases [16] but not yet for malignant neoplasms. Eculizumab, a humanized anti-C5 antibody, has been approved by the Food and Drug Administration (FDA) since 2007 for treating paroxysmal nocturnal hemoglobinuria and has a well-established safety profile. Ravulizumab is a newer anti-C5 antibody with a longer half-life. Furthermore, inhibitors targeting C5a and the C5a receptor (C5aR) have been developed. These include the anti-C5a antibody vilobelimab, which received FDA approval in 2023 for treating hospitalized adults with COVID-19, as well as C5aR inhibitors such as the small molecules ACT-1014-6470 and ALS-205, and the anti-C5aR antibody avdoralimab [17].

## Conclusions

In summary, the available evidence suggests that further research into the pathophysiological mechanisms of anaphylatoxin-NSCLC interactions is essential, given its significant potential for developing novel therapies and biomarkers. Although several complement inhibitors targeting C5a or C5aR1 are available, more clinical trials are necessary to validate their efficacy and applicability for treating NSCLC and other cancers where complement mediators contribute to tumor progression. Future research should prioritize large-scale clinical studies with standardized biomarker measurements, as well as clinical trials evaluating complement-targeted therapies in well-defined NSCLC patient populations.
